# Supplementary material for: Prevention of Musculoskeletal Diseases and Pain among Dental Professionals through Ergonomic Interventions: A Systematic Literature Review
Source: Int J Environ Res Public Health. 2020 May 16;17(10):3482. doi: 10.3390/ijerph17103482 (PMC7277669; doi:10.3390/ijerph17103482)
Supplement: Supplementary file 1 [file ijerph-17-03482-s001.zip › Appendix_S1.pdf]

# Appendix_S1

1 Dent*

2 Dental personnel/ professionals

3 Oral health

4 1 OR 2 OR 3

5 Ergonomics/ human engineering

6 Intervention

7 (Primary) prevention

8 5 OR 6 OR 7

9 Musculoskeletal diseases

10 Musculoskeletal pain

11 9 OR 10

12 4 AND 8 AND 11

13 Limit 12 to yr=”2008-current”

14 Limit 13 to lang=”English”

15 Limit 14 to “abstract available”

16 Limit 15 to “humans”
