# Supplementary material for: Prevention of Musculoskeletal Diseases and Pain among Dental Professionals through Ergonomic Interventions: A Systematic Literature Review
Source: Int J Environ Res Public Health. 2020 May 16;17(10):3482. doi: 10.3390/ijerph17103482 (PMC7277669; doi:10.3390/ijerph17103482)
Supplement: Supplementary file 1 [file ijerph-17-03482-s001.zip › Appendix_S2.pdf]

Appendix_S2

Number	Criterion	Item	Answer option
1	Reporting	Is the hypothesis/aim/objective of the study clearly described?	Yes; no
2		Are the main outcomes clearly described in the Introduction or Methods section?	Yes; no
3		Are the characteristics of the subjects included in the study clearly described?	Yes; no
4		Are the interventions clearly described?	Yes; no
5		Are the distributions of principal confounders clearly described?	Yes; partially; no
6		Are the main findings of the study clearly described?	Yes; no
7		Does the study provide estimates of the random variability in the data for the main findings?	Yes; no
8		Have all important adverse events that may be a consequence of the intervention been reported?	Yes; no
9		Have the characteristics of subjects lost to follow-up been described?	Yes; no
10		Have (actual) probability values been reported for the main findings?	Yes; no
11	External validity	Were the subjects asked to participate in the study representative of the entire population from which they were recruited?	Yes; no; unable to determine
12		Were those subjects who were prepared to participate representative of the entire population from which they were recruited?	Yes; no; unable to determine
13		Were the staff, places and facilities representative?	Yes; no; unable to determine
14	Internal validity - bias	Was an attempt made to blind subjects to the intervention?	Yes; no; unable to determine
15		Was an attempt made to blind study managers?	Yes; no; unable to determine
16		If applicable, was „data dredging" made clear?	Yes; no; unable to determine
17		Do the analyses adjust for different lengths of follow-up of subjects?	Yes; no; unable to determine
18		Were the statistical tests used to assess the main outcomes appropriate?	Yes; no; unable to determine
19		Was compliance with the intervention reliable?	Yes; no; unable to determine
20		Were the main outcome measures used valid and reliable?	Yes; no; unable to determine

21	Internal validity - confounding	Were the subjects in different intervention/control groups recruited from the same population?	Yes; no; unable to determine
22		Were the subjects in different intervention/control groups recruited over the same period of time?	Yes; no; unable to determine
23		Were the subjects randomized to intervention groups?	Yes; no; unable to determine
24		Was the randomized intervention assignment concealed from both subjects and study managers until recruitment was complete and irrevocable?	Yes; no; unable to determine
25		Was there adequate adjustment for confounding in the analyses of the main findings?	Yes; no; unable to determine
26		Were losses of subjects to follow-up taken into account?	Yes; no; unable to determine
27	Power	Did the study have sufficient power to detect a clinically important effect?	0-5 points range
			**Σ 32 points**

Classification of study quality defined by the authors: 0-10 points = low quality; 11-21 points = moderate quality; 22-32 points = high quality.

Reference: Downs & Black (1998): The feasibility of creating a checklist for the assessment of the methodological quality both of randomized and non-randomized studies of health care interventions. J Epidemiol Community Health 52; 377-384.
